# Plasmid-mediated AmpC β-lactamase (CMY-2) gene in *Salmonella* typhimurium isolated from diarrheic pigs in South Korea

**DOI:** 10.1186/1756-0500-7-329

**Published:** 2014-06-02

**Authors:** Ki-Eun Lee, Seong-In Lim, Hwan-Won Choi, Suk-Kyung Lim, Jae-Young Song, Dong-Jun An

**Affiliations:** 1 Animal and Plant Quarantine Agency, Anyang, Republic of Korea; 2 ChoongAng Vaccine Laboratories, Daejon, Republic of Korea

**Keywords:** *Salmonella* typhimurium, Pig, Plasmid, PABL, CMY-2

## Abstract

**Abstracts:**

**Background**

*Salmonella* resistant to third-generation cephalosporin has been isolated from an increasing number of animals worldwide. The purpose of this study was to examine ESBL (extended-spectrum β-lactamases)-producing and PABL (plasmid-mediated AmpC β-lactamases)-producing *Salmonella* isolates from pigs in South Korea.

**Results**

*Salmonella* Typhimurium KVCC-BA1300259 was resistant to ampicillin, amoxicillin/clavulanic acid, cephalothin, chloramphenicol, florfenicol, cefoxithin, gentamicin, nalidixic acid, trimethoprim/sulfamethoxazole, tetracycline, and ceftiofur. The results of a double-disk synergy test and PCR confirmed that the isolate produced CMY-2 (PABL). Analysis of plasmid incompatibility (Inc) groups revealed the presence of IncA/C and IncFIB, indicating antimicrobial resistance. This study is the first to identify *S.* Typhimurium isolates harboring CMY-2 in pigs in South Korea.

**Conclusions**

The presence of CMY-2 in pigs poses a significant threat of possible horizontal spread between animals and humans.

## Background

*Salmonella* sp. are important zoonotic pathogens, are widespread, and can colonize or infect a variety of domesticated and wild animals, including mammals, birds, and reptiles [1-3]. In pigs, Salmonellosis is an infectious digestive disease, which presents with acute or chronic symptoms. *Salmonella* Choleraesuis and *Salmonella* Typhimurium are the two main causative agents of salmonellosis worldwide [4]; however, *S.* Typhimurium is the main cause of disease in pigs in Korea [5].

Since cephalosphorin was developed as an antimicrobial agent, an expanded-spectrum cephalosphorin is recommended for the treatment of salmonellosis [6]. However, *Salmonella* can produce β-lactamase, which digests third-generation cephalosphorins and renders them ineffective [7,8]. Antimicrobial resistance to cephalosphorin is conferred by extended-spectrum β-lactamases (ESBL) and plasmid-mediated AmpC β-lactamases (PABL) [9]. ESBL-producing *Salmonella* isolates produce CTX-M, TEM, OXA or SHV-derived ESBL [6,8,10,11]. Recently, *Salmonella* has developed resistance to cephalosporin through the transmission of PABL [12], of which CMY-2 is the most common. CMY-2 was first reported in the USA and is the most widely distributed PABL, with cases also reported in France, Germany, Greece and the United Kingdom; indeed, it was recently isolated from a cow in Japan and from pigs in China [1,3,12-14]. In most cases, the CMY-2 gene is present in large plasmids, of which several genetic types have been reported. Because it is encoded within a plasmid, CMY-2 can be transmitted horizontally. Thus, there is increasing concern that PABL may spread among pathogens circulating in animals and humans [6].

Here, we isolated CMY-2-producing *S.* Typhimurium isolates from diarrheic pigs in South Korea, and examined the potential horizontal transmission of PABL determinants through plasmids.

## Methods

### Isolation and identification of *Salmonella*

Porcine fecal samples were collected from livestock with digestive disease, such as diarrhea and enteritis by Choong-Ang Vaccine Laboratories of animal appraisal organization. A total of 483 diarrheal fecal samples were obtained from nine provinces (Gyeonggi, Gangwon, Chungbuk, Chungnam, Jeonbuk, Jeonnam, Gyeongbuk, Gyeongnam, and Jeju) in South Korea from June 2011 to June 2012. Samples were mixed with 45 ml of buffered peptone water and incubated for 20 h at 37°C. After incubation, 0.1 ml of sample was inoculated into 10 ml Rappaport Vassiliadis R10 broth (RV, Merck, Germany) and then incubated for 24 h at 42°C. One loop of RV culture was streaked onto the surface of XLD agar (Difco, USA) and Salmonella-Shigella agar (Difco) plates and the suspected colonies were serotyped using Salmonella antisera (Denka Seiken, Tokyo, Japan) according to the method of Ewing [4]. Isolates of *Salmonella* sp. were deposited in the Korea Veterinary Culture Collection (KVCC), where they were stored at -70°C until further use.

### Measurement of minimal inhibitory concentrations and double-disk synergy tests

Minimal inhibitory concentrations (MICs) were determined using the standard broth dilution methods described in Clinical and Laboratory Standard Institute (CLSI) guidelines. *Escherichia coli* ATCC 25922 was used as a control strain. The double-disk synergy test (DDST), which is used to detect β-lactamases, was performed with either 30 μg cefotaxime or 30 μg ceftazidime alone, or with either 30 μg cefotaxime or 30 μg ceftazidime plus 10 μg clavulanic acid according to CLSI guidelines. The DDST was considered positive when the inhibition zone produced by the combined effects of either ceftazidime or cefotaxime plus clavulanic acid was ≥5 mm larger than that produced by either ceftazidime or cefotaxime alone.

### Amplification and sequencing of *β*-lactamases

Multiplex PCR to detect ESBL or PABL genes was performed as described previously [1]. DNA fragments were separated on a 1% agarose gel. Fragments of the appropriate size [1] were extracted from the gel and purified using a Gel Extraction kit (Qiagen Inc., CA, USA), followed by sequencing in an ABI Prism 373 Genetic Analyzer (Applied Biosystems, Foster City, USA) using Sanger’s method. A database search was then performed using the BLAST program at NCBI (http://www.ncbi.nlm.nih.gov).

### Conjugation testing

Conjugation with a sodium azide-resistant recipient, *Escherichia coli* J53, was performed using broth methods [9]. Conjugation strains were selected by plating on MacConkey agar containing 64 mg/L of ceftazidime and 128 mg/L of sodium azide.

### Plasmid analysis

Plasmid DNA was purified using a Plasmid mini purification kit (Qiagen Inc., CA, USA). The BAC-Tracker supercoiled DNA ladder (Epicentre Biotechnologies Inc., WI, USA) was used as a size marker for plasmid analysis. Plasmids were analyzed using the PCR-based replicon typing method to identify the plasmid type [15]. All detected replicon types were confirmed by sequencing.

## Results

Forty-four *Salmonella* sp were isolated from 483 diarrhea samples. Of these, 35 strains were serotyped as Typhimurium (*S.* Typhimurium). The standard broth dilution method was used to determine the antimicrobial susceptibility of *S.* Typhimurium. The antimicrobial susceptibility of the 35 *S.* Typhimurium strains is shown in Table 1. Two strains (*S.* Typhimurium KVCC-BA1300259 and *S.* Typhimurium KVCC-BA1300271) were resistant to ampicillin, amoxicillin/clavulanic acid, cephalothin, chloramphenicol, florfenicol, cefoxithin, gentamicin, nalidixic acid, trimethoprim/sulfamethoxazole, tetracycline, and ceftiofur (data not shown). However, *S.* Typhimurium KVCC-BA1300259 (isolated in the Chungnam region) was positive in the DDST, with the zone of inhibition for ceftazidime plus clavulanic acid being ≥5 mm larger than that for ceftazidime alone. Thus, KVCC-BA1300259 was classified as an ESBL-producer. Moreover, genetic analysis revealed that this isolate produced ESBL and β-lactamase. PCR with primers specific for CMY-2 amplified an 856 bp DNA fragment. Sequence analysis of the CMY-2 gene revealed 100% homology with the *Salmonella* plasmid CMY-2 AmpC beta-lactamase gene (GenBank accession no. JN714983).

**Table 1:** **Antimicrobial susceptibility of *Salmonella *** **typhimurium isolated from diarrheic pigs**

**Antimicrobial agent**	***Salmonella *** **typhimurium (n = 35)**		
	**S**^**a **^**(%)**	**I (%)**	**R (%)**
Ampicillin	22.9	-	77.1
Amoxicillin/clavulanic acid	88.6	5.7	5.7
Cephalothin	68.6	17.1	14.3
Chloramphenicol	37.1	2.9	60.0
Florfenicol	28.6	11.4	60.0
Cefoxitin	88.6	5.7	5.7
Gentamicin	28.6	2.8	68.6
Nalidixic acid	28.6	-	71.4
Streptomycin^b^			
Trimethoprim/sulfamethoxazole	65.7	-	34.3
Tetracycline	11.4	-	88.6
Ceftiofur	91.4	2.9	5.7

^a^S, susceptible; I, intermediate; R, resistant.

^b^No CLSI guidelines.

Antimicrobial resistance of KVCC-BA1300259 was transferred to recipient *E. coli* J53 by conjugation. The KVCC-BA1300259-TC (transconjugant) was resistant to chloramphenicol, gentamicin, streptomycin, tetracycline, ampicillin, amoxicillin/clavulanic acid, cefoxitin, ceftiofur, and cephalothin (Table 2). PCR detected CMY-2 genes in both KVCC-BA1300259 and KVCC-BA1300259-TC (transconjugant).

**Table 2:** **Minimum inhibitory concentrations, plasmid replicon types, and β-lactamase genes expressed by *Salmonella *** **typhimurium isolated from pigs**

**Strain**	**Minimum inhibitory concentration (μg/ml)**												**Plasmid size (kb)/β-lactamase**	**Replicon type**
	**AMP**	**AUG**	**CEP**	**CHL**	**FFN**	**FOX**	**GEN**	**NAL**	**STR**	**SXT**	**TET**	**XNL**		
*S. typhimurium* KVCC-BA1300259	>64	>64/32	>64	>64	>64	>32	>32	>128	>128	>4/76	>128	>8	18-25^CMY-2^	IncA/C IncFIB
*S. typhimurium* KVCC-BA1300259-TC	>64	>64/32	>64	>64	>64	>32	>32	4	>128	<0.1/2.3	>128	>8	18-25^CMY-2^	IncA/C IncFIB
*E.coli* J53 Azide^r^	8	16/8	32	4	<2	4	<1	4	8	<0.1/2.3	<2	<0.5		
*E.coli* ATCC25922	4	4/2	16	4	<2	2	<1	<2	4	<0.1/2.3	<2	<0.5		

AMP: ampicillin; AUG: amoxicillin/clavulanic acid; CEP: cephalothin; CHL: chloramphenicol; FFN: florfenicol; FOX: cefoxitin; GEN: gentamicin; NAL: nalidixic acid; STR: streptomycin; SXT: trimethoprim/sulfamethoxazole; TET: tetracycline; XNL: ceftiofur.

TC: transconjugant.

Azide^r^: sodium azide resistant.

*S.* Typhimurium KVCC-BA1300259 and KVCC-BA1300259-TC harbored a common plasmid ranging from 18 kb to 25 kb in size, and PCR-based plasmid typing identified the incompatibility (Inc) type of this plasmid as IncA/C and IncFIB (Table 2).

## Discussion

Ceftiofur, which was developed strictly for veterinary use, is used throughout the world to treat diseased livestock [7]. However, animal infection by ESBL-producing and PABL-producing *Salmonella* has increased worldwide. It is thought that these bacteria emerged in response to the over-use of ceftiofur [6,16].

One hundred and sixty-five *Salmonella* sp strains were isolated from cattle in China between 2010 and 2011. Of these, 25 strains harbored *β*-lactamases. OXA-1 was the most commonly identified β-lactamase gene (n = 14), followed by TEM-1 (n = 6), PSE-1 (n = 4), and CMY-2 (n = 1) [1]. A study of 283 *Salmonella* sp isolated from Korean chickens between 2002 and 2010 showed that 17 of the ceftiofur-resistant isolates were positive for genes encoding CTX-M-14 and CTX-M-15 [9]. Another study found that two *S.* Typhimurium strains isolated from cattle in Japan harbored both TEM-1 and CMY-2 [14]. Plasmid-mediated AmpC-β-lactamases are frequently identified in human *Salmonella* isolates in South Korea [17]; however, until now, CMY-2 has not been isolated from cattle or pigs. The present study is the first to report the isolation of CMY-2-producing *S.* Typhimurium from pigs in South Korea. The potential spread of CMY-2-producing *S.* Typhimurium via food, particularly animal-derived foods, has important public health implications because CMY-2 is usually plasmid-encoded.

These plasmids can be classified according to size, composition, and incompatibility (Inc) type, and by plasmid multilocus sequence typing [12,14,18]. More recently, the Inc type has been used to classify plasmids. This method is an important tool for tracking the diffusion of plasmids conferring antimicrobial resistance [15]. Of the different Inc types, both IncI1 and IncA/C plasmids were common carriers. The IncI1 plasmid only carries the CMY resistance determinant, whereas the IncA/C plasmids carry at least one additional determinant. The IncA/C plasmids carry genes that confer resistance to at least four antimicrobial agents: chloramphenicol, gentamicin, streptomycin, and tetracycline [12,14].

Plasmids can be horizontally transmitted between bacterial populations via conjugation or mobilization. CMY *β*-lactamase-encoding plasmids harbored by human *Salmonella* isolates in the USA tended to be either large MDR IncA/C plasmids or IncI1 plasmids harboring a single resistance determinant [12]. The IncA/C and IncI1 plasmids were the most common CMY-2 replicon type identified in human *Salmonella* isolates in Spain between 2001 and 2005 [6]. The plasmid replicon types of CMY-2 *β*-lactamase-producing *S.* Typhimurium isolated from a cow in Japan were IncI1 and IncA/C [14]. However, the plasmids identified in the present study were IncA/C and IncFIB. IncFIB was a single chimeric plasmid containing more than one replication type.

Adding antimicrobial agents to animal feed was prohibited in South Korea in July 2011. In the light of the new regulations, continuous monitoring of antimicrobial susceptibility in strains isolated from livestock is warranted due to the increasing prevalence of antimicrobial resistance.

## Conclusion

*S.* Typhimurium isolates from livestock pigs in South Korea harbored CMY-2, implying the potential transfer of antimicrobial resistance. This finding suggests that plasmids harboring CMY-2 pose a significant threat of horizontal transmission between animals and humans.

## Competing interests

The authors declare that they have no competing interests.

## Authors’ contributions

All authors read and approved the manuscript. All authors contributed to the writing of the paper. KL was primarily responsible for collecting the samples and performing the laboratory tests.
